# FIONA1-mediated methylation of the 3’UTR of *FLC* affects *FLC* transcript levels and flowering in Arabidopsis

**DOI:** 10.1371/journal.pgen.1010386

**Published:** 2022-09-27

**Authors:** Bin Sun, Kaushal Kumar Bhati, Peizhe Song, Ashleigh Edwards, Louise Petri, Valdeko Kruusvee, Anko Blaakmeer, Ulla Dolde, Vandasue Rodrigues, Daniel Straub, Junbo Yang, Guifang Jia, Stephan Wenkel

**Affiliations:** 1 Department of Plant and Environmental Sciences, University of Copenhagen, Frederiksberg, Denmark; 2 Copenhagen Plant Science Centre, University of Copenhagen, Frederiksberg, Denmark; 3 Synthetic and Functional Biomolecules Center, Beijing National Laboratory for Molecular Sciences, Key Laboratory of Bioorganic Chemistry and Molecular Engineering of Ministry of Education, College of Chemistry and Molecular Engineering, Peking University, Beijing, China; 4 Quantitative Biology Center (QBiC), University of Tübingen, Auf der Morgenstelle, Tübingen, Germany; 5 NovoCrops Center, Frederiksberg, Denmark; Max Planck Institute of Molecular Plant Physiology: Max-Planck-Institut fur molekulare Pflanzenphysiologie, GERMANY

## Abstract

Adenosine bases of RNA can be transiently modified by the deposition of a methyl-group to form N^6^-methyladenosine (m^6^A). This adenosine-methylation is an ancient process and the enzymes involved are evolutionary highly conserved. A genetic screen designed to identify suppressors of late flowering transgenic Arabidopsis plants overexpressing the miP1a microProtein yielded a new allele of the FIONA1 (FIO1) m^6^A-methyltransferase. To characterize the early flowering phenotype of *fio1* mutant plants we employed an integrative approach of mRNA-seq, Nanopore direct RNA-sequencing and meRIP-seq to identify differentially expressed transcripts as well as differentially methylated RNAs. We provide evidence that FIO1 is the elusive methyltransferase responsible for the 3’-end methylation of the *FLOWERING LOCUS C* (*FLC*) transcript. Furthermore, our genetic and biochemical data suggest that 3’-methylation stabilizes *FLC* mRNAs and non-methylated *FLC* is a target for rapid degradation.

## Author summary

Plants constantly measure environmental parameters such as temperature, light-quality, -quantity and -duration, as well as other parameters to adjust their growth and development. The transition to flowering is a crucial transition during plant development because it impacts the number of fertile offspring a plant can produce. Thus, this transition is under tight control by various transcription factors and epigenetic mechanisms. In a genetic screen for early flowering mutants, we identified the gene encoding the FIONA1 methyltransferase to act as a repressor of flowering. Investigation of the changes of the transcriptome and m^6^A-RNA-methylome revealed a large number of differentially expressed genes and methylated RNAs. Among these RNAs is the also the mRNA of the floral repressor *FLOWERING LOCUS C* that shows loss of m^6^A methylation in the 3’UTR that causes destabilization of the *FLC* transcript.

## Introduction

Modification of RNA is pervasive and found across the entire tree of life [1]. In mRNA, *N6*-methyladenosine (m^6^A) is the most abundant internal covalent modification. m^6^A methylation patterns in plant mRNA have been found to be conserved between distant ecotypes [2] suggesting ancient regulatory functions. Biochemical studies have revealed that the mammalian m^6^A -writer complex consists of METTL3, METTL14, and associated proteins, such as WTAP and the ubiquitin ligase HAKAI [3–5]. Besides METTL3 and METTL14, METTL16 is a U6 adenosine methyltransferase that has been implicated in controlling m^6^A -methylation of mRNAs in humans [6] and has been shown in worms to affect diet-induced splicing of mRNA transcripts [7]. In plants, the functions of homologs of METTL3 and METTL14, MTA and MTB, respectively, as m^6^A -methylation writers are well characterized [4, 5]. For example, it is known that loss of MTA causes embryonic arrest at the globular stage [4], demonstrating the biological importance of m^6^A. In addition to m^6^A -writers, m^6^A readers, i.e. RNA binding proteins with specificity for m6A, can recognize m^6^A marks and affect RNA stability, splicing and translation through an unknown molecular mechanism [8]. The analysis of an early flowering knock-down allele of the METTL16-homolog FIONA1, *fio1-2*, revealed changes in the m^6^A methylation status of many transcripts, several encoding flowering regulators including *SUPPRESSOR OF OVEREXPRESSION OF CONSTANS* (*SOC1*) [9]. Besides *SOC1* mRNA, the mRNA of the flowering regulator *FLOWERING LOCUS C* (*FLC*) has also been shown to be modified by m^6^A -methylation [10]. The latter study showed that an R-loop forms at the FLC locus that is resolved by the RNA-binding proteins FCA and FY. In this process, FCA binds the *FLC COOLAIR* antisense transcript to facilitate m^6^A -methylation [10]. Interestingly, the authors also detected m^6^A -methylation of the 3’UTR of *FLC* mRNA that appeared to be installed independently of FCA.

Here, we isolated a novel allele of *FIONA1* (*FIO1*) in a genetic screen for suppressors of the late flowering phenotype of plants overexpressing the miP1a microProtein [11]. We present evidence that FIO1 acts as an m^6^A -methyltransferase in Arabidopsis and is the functional homolog of human METTL16. Using a combination of mRNA-seq, meRIP-seq and Nanopore direct RNA-sequencing, we provide further evidence that FIO1 is the elusive 3’UTR methyltransferase of *FLC*. Moreover, our data shows that the largely pleiotropic phenotype of *fio1* mutant plants is a result of massive transcriptome and RNA-methylome changes. In the case of *FLC*, FIO1 is needed to maintain 3’-end methylation. Abrogation of this methylation mark causes depletion of *FLC* mRNA.

## Results

### FIONA1 acts as a floral repressor that functions partially independent of the photoperiod pathway

**Fig 1:**
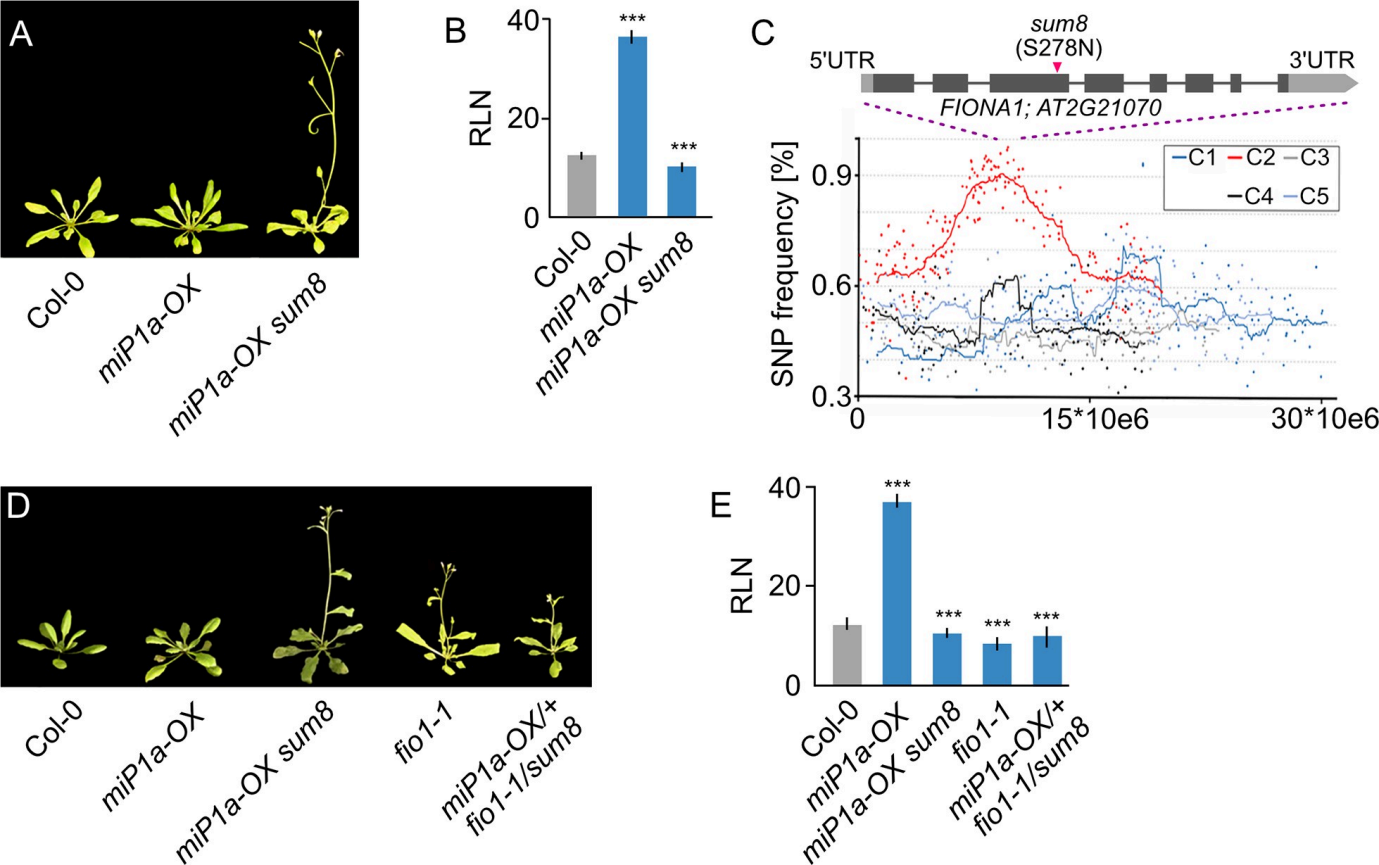
Identification of the flowering repressor FIONA1 by whole-genome re-sequencing. **(A)** Phenotype of the *sum8* (*fio1*) mutant in the miP1a-OX background compared to the Col-0 wildtype grown in LD conditions. **(B)** Determination of flowering by counting the number of rosette leaves (RLN = rosette leaf number) at the bolting stage in LD. Plotted are average leaf number +/- SD, ***p = <0.001, N = 10. **(C)** Mapping-by-sequencing of the *sum8* suppressor mutation. Plotted are SNP frequencies of a pool of segregating F2 plants. Increased SNP frequencies were observed in chromosome 2 and the FIO1 locus is at the summit of the plot. **(D)** Genetic complementation experiment proving that the *sum8* mutation affects *FIO1*. Shown are the flowering phenotypes of plants grown in LD conditions. **(E)** Determination of flowering by counting the number of rosette leaves (RLN = rosette leaf number) at the bolting stage in LD. Plotted are average leaf number +/- SD, ***p = <0.001, N = 10.

The miP1a/miP1b microProteins act as suppressors of flowering by interacting with a TOPLESS-containing repressor complex [11, 12]. To identify factors that are required for the repressor complex to suppress flowering, we performed a genetic screen with transgenic *miP1a-OX* (*35S*::*MIP1A)* plants. We identified a set of *su**ppressor of* *m**iP1a* (*sum*) mutants, that, despite high levels of miP1a protein, flowered early under inductive long day conditions [12]. One of the suppressors, *sum8*, we describe here, showed accelerated flowering compared to the non-mutagenized *miP1a-OX* parental plant (Fig 1A and 1B). To identify the causal mutation in the *sum8* background, we crossed *miP1a-OX sum8* plants to Col-0 wildtype, self-pollinated the offspring and selected a pool of 20 BASTA-resistant suppressor mutants of the following generation. Pooled DNA of the *sum8* suppressor mutant and the parental line was then analyzed by genome re-sequencing. In total, we detected 685 EMS-induced SNPs with a frequency enrichment in the middle of chromosome 2 (Fig 1C). At the summit region of the enrichment peak we identified a point mutation in the *FIONA1* (*FIO1*) gene which converted serine 278 into an asparagine (S278N). To verify that the mutation in *FIO1* is causal for the early flowering phenotype, we obtained a second EMS allele (*fio1-1*) that had been described earlier [13] and crossed it with *miP1a-OX sum8* plants. The resultant nullizygote offspring (*miP1a-OX/+ fio1-1/sum8*) flowered early (Fig 1D and 1E), supporting that the mutation in *FIO1* is indeed causal for the flowering phenotype. The *fio1-1* allele is a splice site mutation that results in the loss of five amino acids while *sum8* is a point mutation. To obtain an additional *FIO1* allele, we used a CRISPR approach with multiple sgRNAs and obtained the new *fio1-5* allele. Like *fio1-1* and *sum8*, *fio1-5* also showed early flowering in long day conditions (S1A and S1B Fig). The *fio1-5* deletion occurred close to a splice site and caused the loss of amino acids 53–64 and the conversion of amino acid residues 66–72 (S1C Fig).

### FIO1 is related to the human METTL16 protein

FIO1 is a nuclear localized protein containing a DUF890 domain, making it a member of the METTL16-like protein family that is comprised of, among others, the human and mouse METTL16 and the *C*. *elegans* METT-10 proteins. Animals carrying loss-of-function alleles of METT-10/METTL16 have been described to show severe developmental defects, and sometimes, lethality [14, 15]. The latter finding raised the question of whether we were dealing with complete loss-of-function or reduced function alleles of *FIO1*. All mutants had either smaller deletions or single amino acid changes suggesting they could be weak, reduced function alleles.

To gain further insights into the alleles that we had obtained, we created a homology model of the FIO1 methyltransferase (MTase) domain and compared it against the crystal structure of the human homologue, METTL16. In the case of the *sum8* mutation (S278N, S2 Fig), we found that the sidechain of S278 normally forms hydrogen bonds with the nitrogen on the W330 within the protein core. Upon mutating the serine to an asparagine, we expect that the larger asparagine sidechain cannot be accommodated in the protein interior, leading to disrupted domain fold and function. The *fio1-1* mutation involves the deletion of five amino acids 145–149 in the FIO1 protein (S2 Fig) which includes the disruption of a potential hydrogen bond between the sidechains of Q82 and T147 and the loss of a flexible loop connecting an alpha helix and a beta sheet. The *fio1-5* mutation involves the large deletion of amino acids 57–68 and the non-conservative mutation of residues 53–56 and 69–72 (S2 Fig). Both *fio1-1* and *fio1-5* involve the large-scale disruption of hydrophobic and hydrogen bonding interactions and are likely to result in misfolded or aggregated protein. Thus, it is highly likely that all three mutations (*sum8*, *fio1-1* and *fio1-5*) disrupt the methyltransferase function of FIO1.

**Fig 2:**
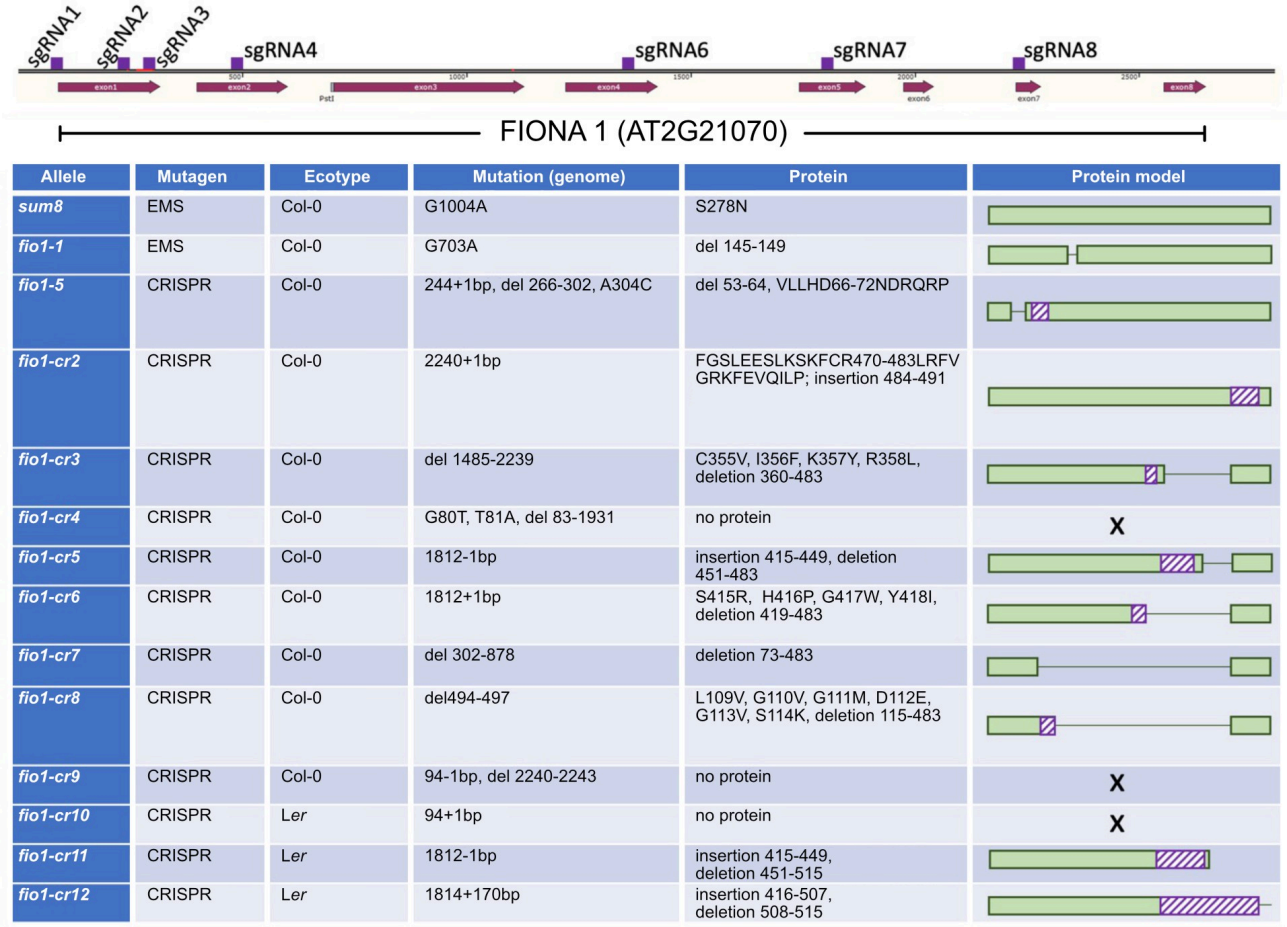
Overview of *fiona1* mutant plants analyzed and generated in this study. Gene model depicting the *FIO1* locus (exons in dark red and location of sgRNAs in purple). All sgRNAs were transformed in bulk and from all early flowering individuals the *FIO1* gene was sequenced to determine the nature of CRISPR-induced mutations.

To validate the findings of the protein modeling we employed a second CRISPR mutagenesis approach and designed eight sgRNAs spanning the entire *FIO1* locus and transformed these in bulk to obtain larger structural mutations (Fig 2). We identified 11 new *FIO1* alleles, of which several had large structural deletions. Three of these alleles (*fio1-cr4*, *fio1-cr9*, *fio1-cr10*) had frame-shift mutations that would not lead to the production of functional proteins. All new alleles were early flowering (S3 Fig) but viable and produced fertile offspring. Taken together, these results show that the loss of METTL16 function is not lethal in plants but affects the transition to flowering.

### The loss of FIO1 function results in a pleiotropic phenotype

**Fig 3:**
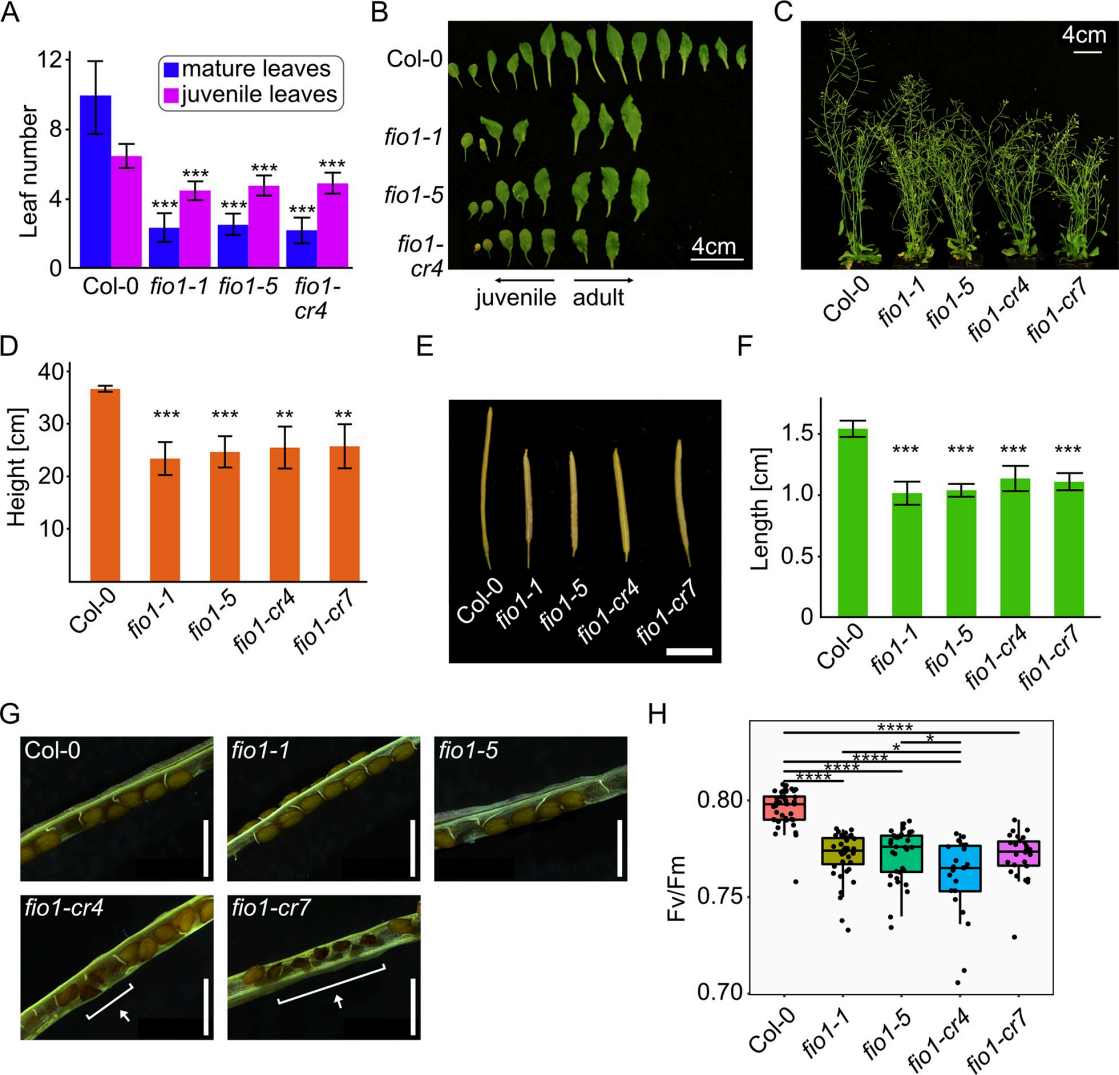
*fio1* mutants display a pleiotropic phenotype. **(A)** Number of mature and juvenile leaves of plants grown under LD at the bolting stage. Plotted are the means with bars denoting +/- SD with N = 11–17. Asterisks represent significance level between values determined by two-sample T-Test. **(B)** Rosette leaf morphology of representative WT and *fio1* mutants at the WT bolting stage. **(C)** Branching phenotype of 44-day old WT and *fio1* mutant plants grown in LD. **(D)** Height of tallest inflorescence of WT and *fio1* mutant plants at senescence. Plotted are the means with bars denoting +/- SD with N = 3–6. Asterisks represent significance level between values determined by two-sample T-Test. **(E)** Silique morphology at maturation. One representative silique from each line. Scale bar is 5 mm. **(F)** Silique length at maturation. Plotted are the means with bars denoting +/- SD with N = 10–12 and 3 biological replicates of the WT and 4 biological replicates per *fio1* mutant line. Asterisks represent significance level between values determined by two-sample T-Test. **(G)** Opened siliques from WT and *fio1* mutants. Highlighted are aborted seeds of *fio1-cr4* and *fio1-cr7*. Scale bar is 1 mm. **(H)** Photosynthetic efficiency of WT and *fio1* mutant seedlings expressed as variable fluorescence/maximum fluorescence (Fv/Fm), N = 23–41. Asterisks represent significance level between values determined by two-sample T-Test. For all plots, *P ≤ 0.05, **P ≤ 0.01, ***P ≤ 0.001, ****P ≤ 0.0001.

Precocious flowering upon loss of *FIO1* function is a striking phenotype that appears early in development. To characterize *fio1* mutants that lack large fractions of the FIO1 protein and compare them to the previously characterized mutants, we recorded the flowering of the panel of novel CRISPR alleles that we generated in this study (Fig 2). This analysis revealed that all *fio1* mutants, regardless of the genetic background (here Col-0 or L*er*), flowered unanimously early (S3 Fig). A more detailed analysis of the *fio1-cr4* mutant that is lacking most of the FIO1 protein, showed that it flowered as early as *fio1-1* and *fio1-5* compared to Col-0 wild type plants. In addition to recording the number of leaves produced at the bolting stage, we also collected all leaves and found that *fio1* mutant plants produce fewer juvenile and mature leaves, indicating that both growth phases (juvenile and adult phase) are accelerated in *fio1* mutants compared to wild type (Fig 3A and 3B). When grown to full maturity, we also detected that *fio1* mutant plants were significantly shorter than wild type plants and also appeared bushier (Fig 3C and 3D). Siliques produced by the *fio1* mutant plants were also notably shorter (Fig 3E and 3F) and we were wondering if this could be due to a higher rate of early seed abortion. A closer inspection of the siliques revealed normal seeds in Col-0 wild type and *fio1-1* and *fio1-5* mutant plants, whereas *fio1-cr4* and *fio1-cr7* plants showed fractions of improperly developed shriveled seeds. The *fio1-cr4* and *fio1-cr7* mutants both carry large structural deletions in the *FIO1* gene and this latter finding suggests that they are both complete loss-of-function alleles. The *fio1-1* and *fio1-5* alleles, on the other hand, might have residual FIO1 enzyme activity or participate in FIO1 protein complexes that do not involve its methyltransferase activity. Besides the smaller stature and earlier flowering, *fio1* mutant plants also appeared paler in color compared to wild type plants. To determine whether *fio1* mutants are additionally impaired in photosynthetic performance, we measured the Fv/Fm ratio using pulse amplitude modulated (PAM) fluorometry. PAM measurements confirmed defects in photosynthetic performance of *fio1* mutant plants and all tested mutants showed consistently lower Fv/Fm ratios (Fig 3H).

### The loss of FIO1 function affects multiple flowering pathways

A previous genetic screen for regulators of flowering resulted in the identification of the *fio1-1* mutant that exhibited early flowering in both long- and short-day conditions [13]. A knock-down mutation caused by a T-DNA insertion in the 5’-region of the *FIONA1* gene [9, 16] showed a similar phenotype. The *fio1-1* mutant was shown to have elevated levels of both *CONSTANS* (*CO*) and *FLOWERING LOCUS T* (*FT*) mRNA. CO is a photoperiod-sensitive transcription factor that accumulates in response to long days to activate *FT* [17], which in turn acts as a florigen to induce flowering [18, 19]. The flowering phenotype of *fio1-1* was ascribed to changes in period length of the central oscillator. Consistent with previous findings, we found that levels of both *CO* and *FT* were elevated in *fio1-1* and *fio1-5* (S4A and S4B Fig). A genetic interaction study revealed that *miP1a miP1b fio1-5* triple mutant plants also flowered early like *fio1-5* mutant plants. The combination of *fio1* mutants with either *co* and *ft* mutants as in *fio1 co* and *fio1 ft*, revealed a promotion of flowering (S4C and S4D Fig) in both short days and long days. These results unequivocally show that the function of FIO1 is independent of the function of miP1a and partially independent of the photoperiod flowering time pathway.

### Transcriptome analysis of *fio1-1* and *fio1-5* mutant plants reveal substantial gene expression changes

**Fig 4:**
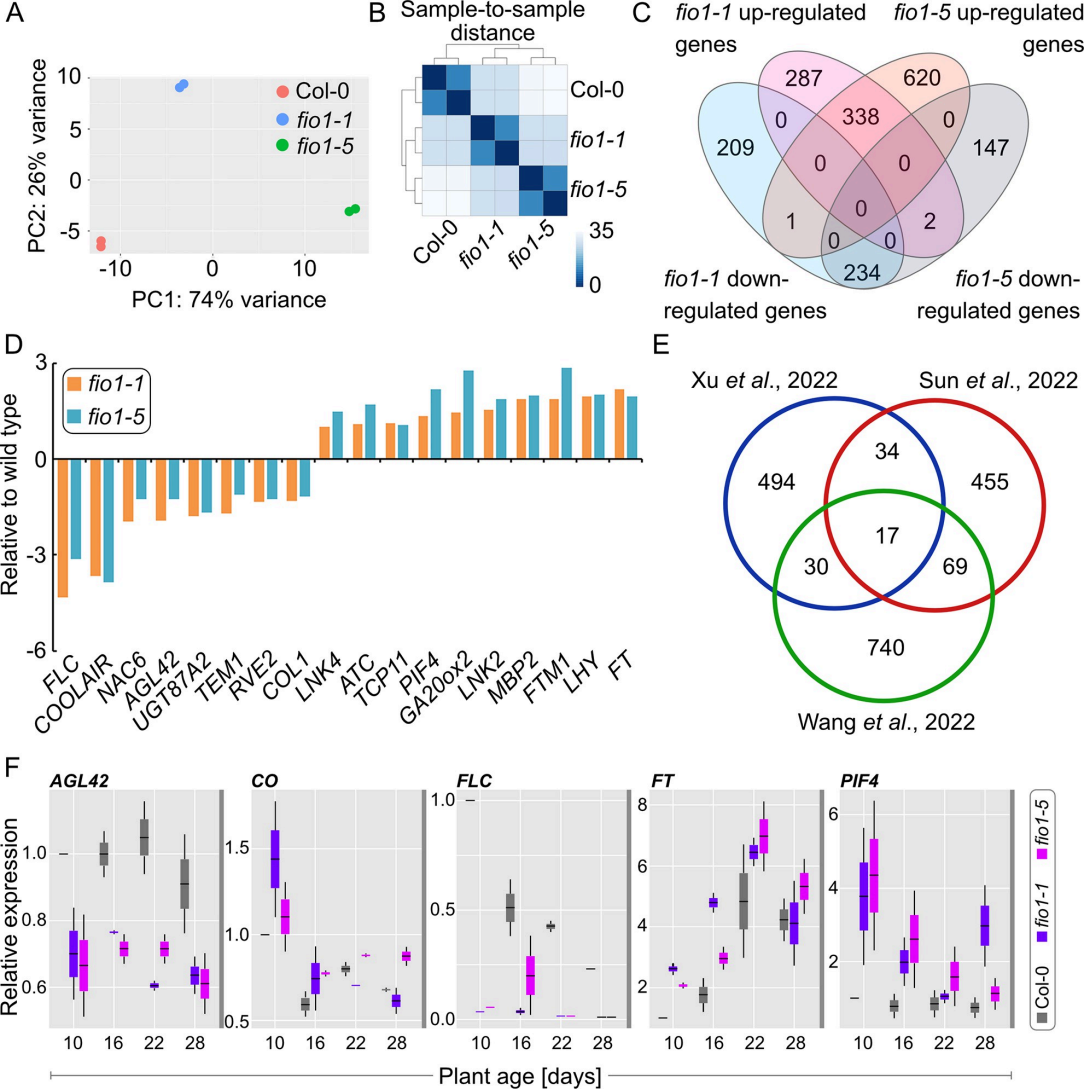
Transcriptome changes observed in *fio1* mutants. **(A)** Principal component analysis (PCA) plot displaying the different RNA-seq performed using DESeq2 rlog-normalized RNA-seq data. Plotted is the percentage of variance for each component. **(B)** Hierarchical clustering analysis (HCA) of the different RNA-seq libraries. The heatmap was built using the DEseq2 package. Samples were clustered using HCA performed with DESeq2 rlog-normalized RNA-seq data, and the dendrogram represents the clustering results. The heatmap illustrates the pairwise distances between the different samples, with higher similarity indicated by higher intensity of color. **(C)** Venn diagram showing the overlap of differentially expressed genes in *fio1-1* and *fio1-5* compared to the wild type. The absolute value of log2 FC (fold change; *fio1* mutant / WT) ≥ 1.0 and adjusted P-value (false discovery rate; FDR) ≤ 0.05. **(D)** RNA-seq showing the expression levels of flowering related genes in *fio1-1* and *fio1-5* compared to the wild type. The absolute value of log2 FC (fold change; *fio1* mutant / WT) ≥ 1.0 and adjusted P-value (false discovery rate; FDR) ≤ 0.05. **(E)** Comparative analysis of differentially expressed genes identified by RNA-seq and nanopore-sequencing in three different studies. P-Value < 0.01, FC > 2. The Venn diagram depicts the overlap of the three datasets. **(F)** Gene expression analysis of candidate genes over a developmental time course. Plants were grown in LD conditions and samples were harvested before the end of the long day, 10, 16, 22 and 28 days after germination. Box plots depict relative expression levels of respective genes to the *UBQ10* and *TIP41* gene of two biological replicates with three technical replicates each.

To obtain a better understanding of how FIO1 affects flowering, we performed an RNA-seq experiment with Col-0, *fio1-1* and *fio1-5* mutant plants to identify differentially expressed genes. RNA of two biological replicates of 14 day-old seedlings was isolated and sequenced on an Illumina HiSeq instrument. After removing low-quality reads, an average of 91.47% of the filtered reads was mapped to the *Arabidopsis thaliana* reference genome. Principal component analysis (PCA) and hierarchical cluster analysis (HCA) revealed that the individual biological replicates clustered closely together (Fig 4A and 4B), indicating a high degree of experimental reproducibility. Interestingly, *fio1-1* and *fio1-5* were also distinct from each other and wild type, indicating that although they show a similar flowering phenotype they might differ at the molecular level.

To identify differentially expressed genes (DEGs) in *fio1-1* and *fio1-5* we used limma-voom [20] with a fold change cutoff of 2.0 or more. In total, we identified 627 and 959 up-regulated genes in *fio1-1* plants and *fio1-5* plants respectively (P value < 0.05 and adjusted P value < 0.05; S1 Table). We found 1071 DEGs in *fio1-1* and 1342 DEGs in *fio1-5* with an overlap of 338 up-regulated genes and 234 down-regulated genes (Fig 4C). Of these deregulated transcripts, 18 were associated with regulation of flowering (Fig 4D), including flowering repressors *FLOWERING LOCUS C* (*FLC*) and *TEMPRANILLO1* (*TEM1*) whose mRNA levels were significantly reduced in *fio1* mutant plants and flowering activators such as *PHYTOCHROME INTERACTING FACTOR4* (*PIF4*), *FT* and *LATE ELONGATED HYPOCOTYL* (*LHY*) whose mRNA levels were significantly increased in *fio1* mutant plants (Fig 4D). These findings are in agreement with the early flowering phenotype of *fio1* mutant plants. Comparative analysis of RNAseq data of two recent studies [9, 21] produced only a limited overlap (Fig 4E) which is likely due to differences in growth conditions and stages of development that were used for the analysis. To validate some of the differentially expressed genes that we had identified in our analysis, we tested the expression of *AGL42*, *CO*, *FLC*, *FT* and *PIF4* by qRT-PCR at four timepoints during plant development (Fig 4F). The expression of *AGL42* and *FLC* was consistently low in *fio1* mutants at all growth stages analyzed, while *CO* and *FT* levels were higher in *fio1* mutants only during the early stages of development and not after the floral transition had occurred (Fig 4F). Expression of the floral thermoregulator *PIF4* was increased in *fio1* mutants at all stages that were analyzed. The findings that *PIF4* is deregulated in *fio1* mutants is in line with *fio1* mutants showing defects in the shade avoidance response (S5 Fig). When germinated in white light conditions, *fio1* seedlings develop elongated hypocotyls and in shade elongate even more than wild type seedlings which is indicative of shade hypersensitivity.

### FIO1 acts as m^6^A -methyltransferase and methylates predominantly the 3’UTR of mRNAs

**Fig 5:**
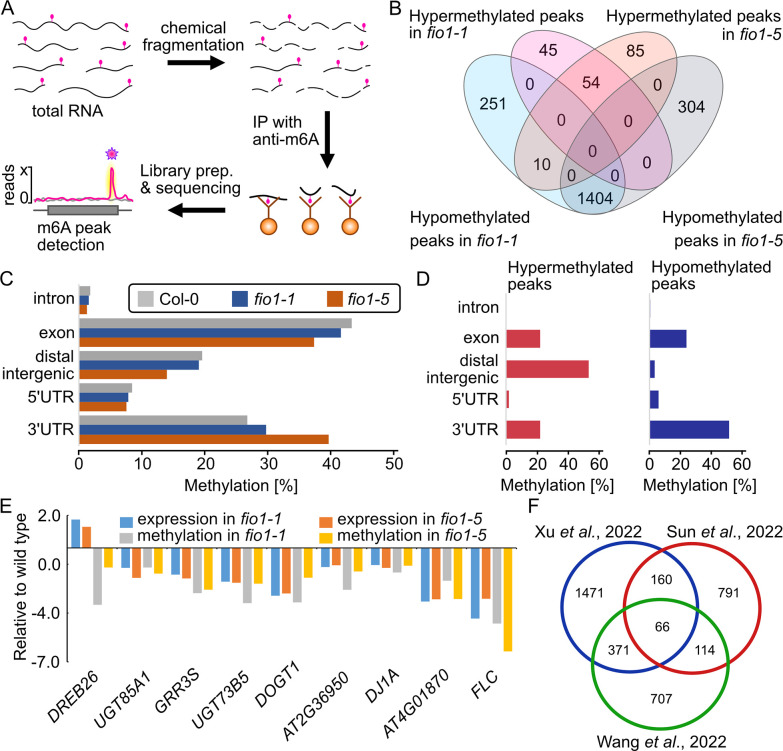
FIONA1 acts as m^6^A-methyltransferase in Arabidopsis. **(A)** Depiction of the meRIP-seq method. In brief, total RNA was isolated from seedlings and subsequently fragmented into small (100bp) fragments. After immunoprecipitation with an m^6^A -specific antibody, Illumina short-read sequencing libraries were generated and sequenced. After mapping all reads to the Arabidopsis genome, m^6^A peak regions (pink star) could be identified. **(B)** Venn diagram showing the overlap of the hypermethylated and hypomethylated m^6^A peaks identified in *fio1-1*, *fio1-5* compared to Col-0 wild type plants. **(C)** Comparison of distribution of m^6^A peaks in different segments of wild-type (left panel), *fio1-1* (middle panel) and *fio1-5* (right panel) transcripts. The panels show pie charts presenting the percentages of m^6^A peaks in different transcript segments. **(D)** Comparison of distribution of m^6^A peaks in different segments of differently methylated peaks (left panel), hypermethylated peaks (middle panel) and hypomethylated peaks (right panel) in the overlap of *fio1-1* and *fio1-5* compared to wild type. The panels show pie charts presenting the percentages of m^6^A peaks in different transcript segments. **(E)** Expression levels and m^6^A methylation levels of the transcripts in the overlapping of RNAseq and MeRIPseq. Gene expression levels were derived from RNA-Seq data. m^6^A methylation levels were derived from MeRIPseq data. **(F)** Comparative analysis of hypomethylated m^6^A peaks in three different studies. MeRIP-seq cutoff RPM>5 and logFC<-0.5; Nanopore cutoffs FC>5 and FDR<0.05. The Venn diagram shows the overlap of the three datasets.

The presence of the DUF890 domain suggests that FIO1 acts as a genuine m^6^A methyltransferase. To identify the FIO1 RNA substrates, we employed a modified version of methylated RNA-immunoprecipitation (meRIP) followed by deep sequencing that was described earlier (Fig 5A) [22]. To determine methylation positions (m^6^A peaks) we used MACS [23] with a false discovery rate (FDR) ≤ 0.05 and enrichment of ≥ 2-fold of sequence reads. In summary, we identified 3,025 m6A-methylation peaks in wild type, 2,088 in *fio1-1* and 2,109 in *fio1-5* (S2 Table). In *fio1-1* plants and *fio1-5* plants we identified 99 and 149 peaks, respectively, with increased m^6^A level compared to wild type. In contrast, a total of 1,665 m^6^A methylation peaks in *fio1-1* and 1,708 peaks in *fio1-5* were decreased or absent compared to the wild type (Fig 5B). These findings suggest that FIO1 methylates mRNAs. When assessing the localization of the m^6^A -peaks globally in wild type, *fio1-1* and *fio1-5*, we observed more peaks in exons of *fio1* mutants and a reduced number of peaks in the 3’UTR of *fio1* mutants compared to wild type (Fig 5C). The differential m^6^A peak distribution analysis (wild type versus *fio1* mutants) revealed a massive over-representation of hypomethylated peaks in 3’UTRs in *fio1* mutants compared to wild type (Fig 5D). These findings indicate that FIO1 acts as m^6^A methyltransferase and methylates the 3’UTRs of its target substrates. To explore a potential connection between m^6^A -methylation and RNA stability we compared our mRNA-seq and MeRIP datasets. In total we found nine genes containing hypomethylated peaks, eight of which were expressed at lower levels while one was expressed at higher level in *fio1* mutants compared to the wild type (Fig 5E). Additional comparative analysis of meRIPseq and nanopore-sequencing data of two recent studies [9, 21] again produced only a limited overlap (Fig 5F) which could indicate differences in growth conditions and stages of development that were used for the analysis.

### FLC is a prime target of FIO1

**Fig 6:**
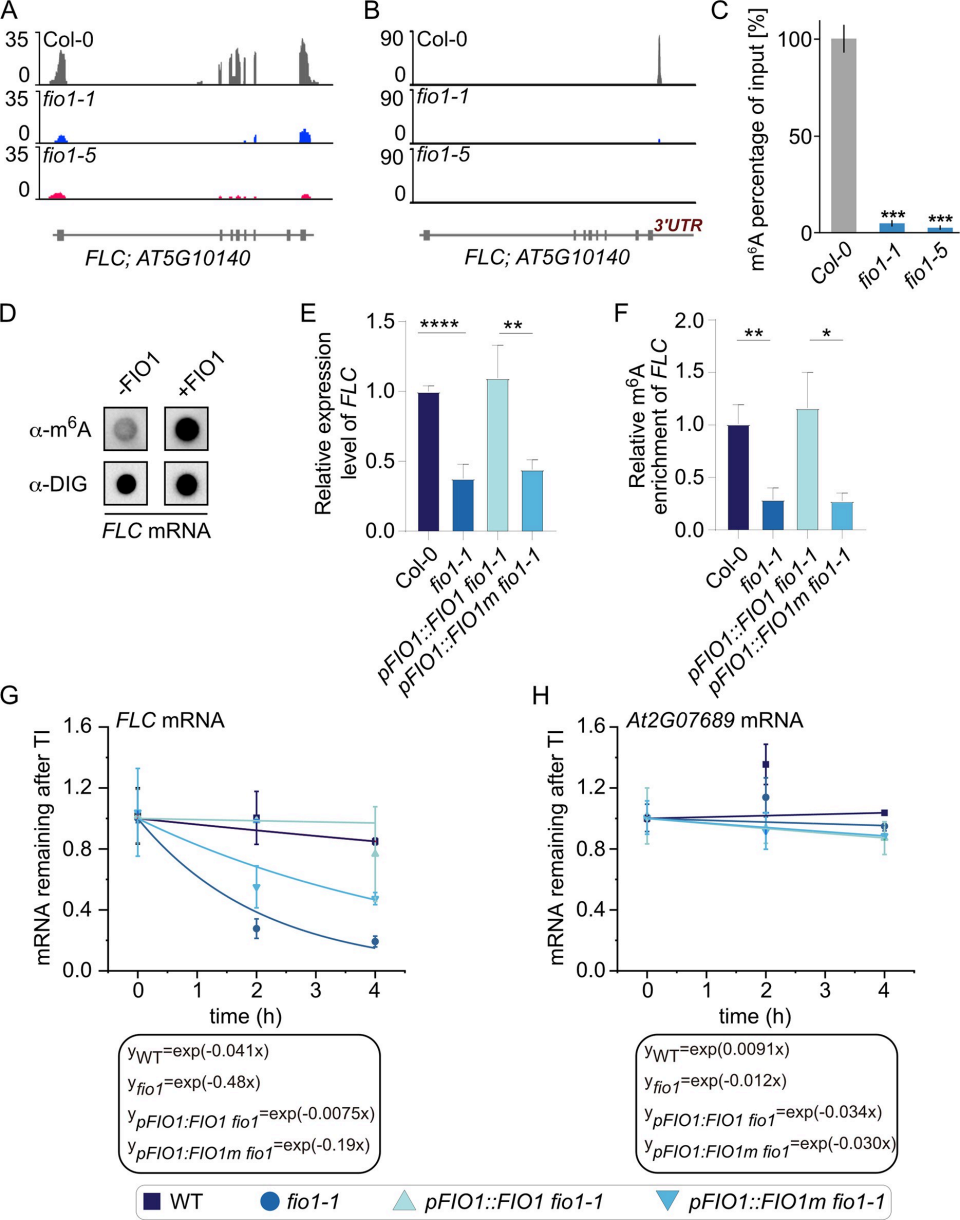
FIONA1 acts as m^6^A-methyltransferase on *FLC*. **(A)** RNA-seq coverage observed at the *FLC* locus. RNA-seq reads in Col-0 (grey), *fio1-1* (blue) and *fio1-3* (pink). Gene model depicts exons and introns. **(B)** MeRIP-seq coverage observed at the *FLC* locus. RNA-seq reads in Col-0 (grey), *fio1-1* (blue) and *fio1-3* (pink). Gene model depicts exons and introns. **(C)** Percentages of the m^6^A methylated FLC mRNA in input samples in the wild type, *fio1-1* and *fio1-3* measured by m^6^A-IP-qRT PCR. Values are the means ±SD. N = 4, ***P≤ 0.001. **(D)** *In vitro* methylation of FLC mRNA. FIO1 was purified as GST-tagged protein from E. coli and incubated with digoxygenin (DIG)-labeled in vitro produced FLC mRNA in the presence of SAM. Dot blot in the upper row show m^6^A methylated signal with the anti- m^6^A antibody. The dots in the lower row the signal of the anti-DIG antibody. Increased signal intensities were detected when FIO1 enzyme was present. **(E)** qPCR results showing the relative expression of *FLC* in 12-day-old Col-0, *fio1*, *pFIO1*:*FIO1/fio1*, *and pFIO1*:*FIO1m/fio1* seedlings. Data are means ± SD for 3 biological replicates × 3 technical replicates. * *p* < 0.05, ** *p* < 0.01 by *t* test (two-tailed). **(F)** m^6^A-IP-qPCR results showing the relative m^6^A levels of *FLC* transcripts in 12-day-old Col-0, *fio1*, *pFIO1*:*FIO1/fio1*, *and pFIO1*:*FIO1m/fio1* seedlings. Data are means ± SD for 3 biological replicates × 3 technical replicates. * *p* < 0.05, ** *p* < 0.01 by *t* test (two-tailed). **(G)** and **(H)** The mRNA lifetimes of *FLC* **(G)** in Col-0, *fio1*, *pFIO1*:*FIO1/fio1*, *and pFIO1*:*FIO1m/fio1*. The *AT2G07689* **(H)** was used as the negative control. TI: transcription inhibition. Data are represented as means ± SD for 2 biological replicates × 3 technical replicates.

The mRNA of the flowering repressor *FLOWERING LOCUS C* (*FLC*) was identified as a prime methylation target of FIO1 (Fig 5E). We detected strongly decreased expression of *FLC* mRNA in *fio1* mutants compared to wild type (Fig 6A) and the m6A peak that can be detected in wild type plants is absent in *fio1-5* and strongly reduced in *fio1-1* mutant plants (Fig 6B). To verify that *FLC* is indeed a *bona fide* methylation target of FIO1, we performed anti- m^6^A antibody immunoprecipitations (m^6^A -IP) of total RNA from wild type (Col-0), *fio1-1* and *fio1-5* seedlings followed by qPCR (m^6^A -IP-qPCR). We found the relative amount of m^6^A methylated *FLC* mRNA was strongly decreased in both *fio1* mutant plants (Fig 6D) confirming that FIO1 is the essential m^6^A methyltransferase that methylates the 3’UTR of *FLC*. To validate if FIO1 is capable of catalyzing the methylation reaction of *FLC* mRNA outside of a cellular context, we purified the Arabidopsis FIO1 enzyme from *E*. *coli* and carried out methylation reactions with *in vitro* synthesized digoxygenin-labelled *FLC* RNA (Fig 6D). In these assays, FIO1 can methylate *FLC* mRNA indicating that the effect is of a direct nature. We also tested the levels of *FLC* mRNA in seedlings and compared it to the enrichment of m^6^A methylation of *FLC* (Fig 6E and 6F). Wild type plants and *fio1* mutants complemented with a transgene expressing *FIO1* from its own promoter have higher levels of both *FLC* mRNA and *FLC* m^6^A methylation (Fig 6E and 6F). This contrasts the findings in *fio1* mutants and *fio1* mutants complemented with a transgene expressing mutant *FIO1m* from its own promoter that have lower levels of *FLC* mRNA and decreased levels of *FLC* m^6^A methylation (Fig 6E and 6F). Quantification of mRNA levels of *FLC* and of the control gene *AT2G07689* after transcriptional inhibition in wild type, *fio1* mutants and previously described complementation lines were also carried out. We found decreased *FLC* mRNA stability in *fio1* and in *fio1* mutants complemented with the non-functional *FIO1m* (Fig 6G). In summary, our data reveal a role for FIO1 as *FLC* methylating enzyme.

### Direct RNA sequencing of wild type and *fio1* mutant plants confirms altered *FLC* mRNA levels in *fio1* mutants

**Fig 7:**
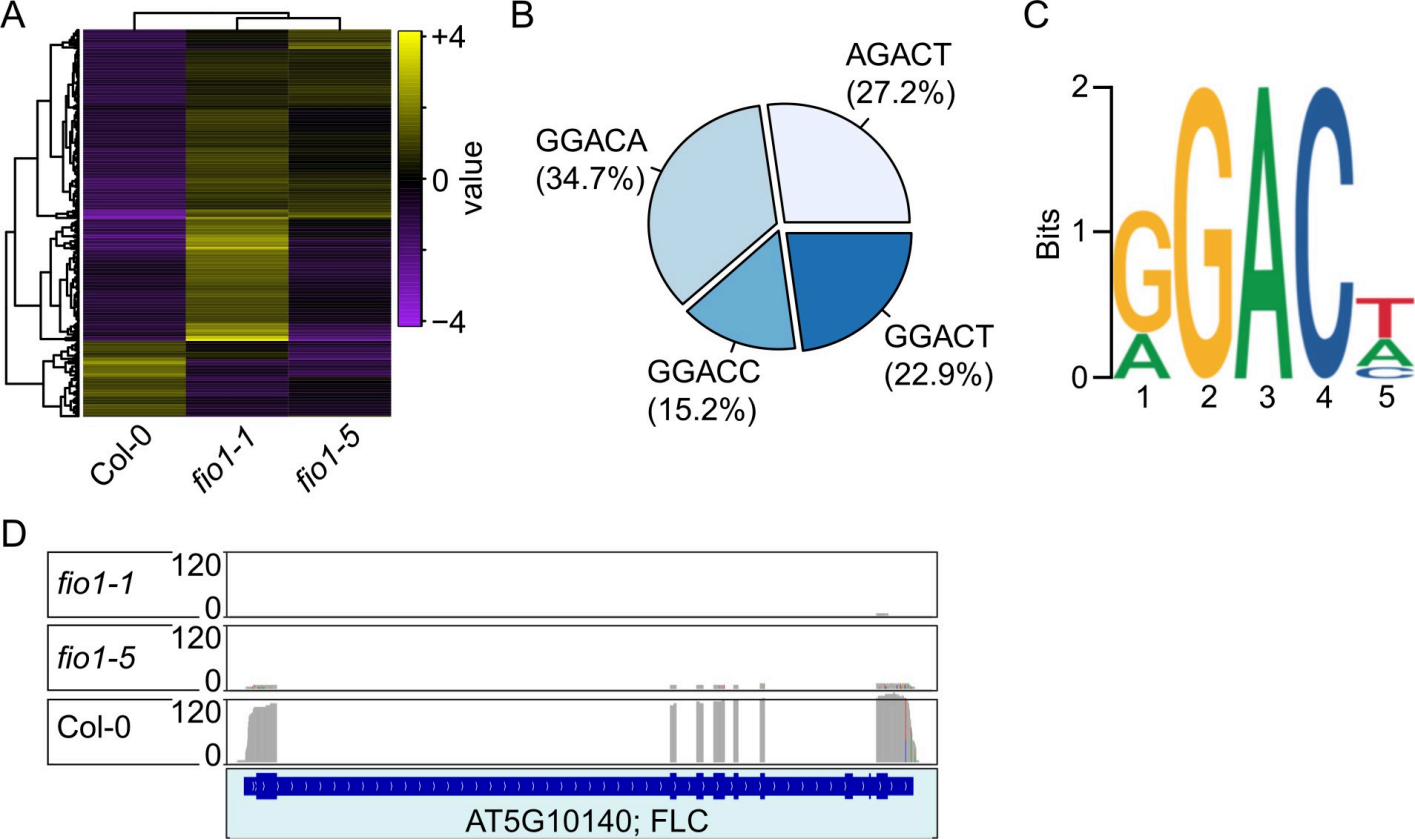
Direct RNA-sequencing analysis. **(A)** Cluster analysis of differentially expressed transcripts in the three different genotypes. **(B)** Distribution of m6A methylations detected by direct RNA-sequencing. **(C)** Logo of the conserved m6A sequence motif detected by direct RNA-sequencing. **(D)** Sequence coverage observed at the *FLC* locus. Direct RNA-seq reads in Col-0, *fio1-1* and *fio1-5*. Gene model depicts exons and introns.

To determine the genome-wide m^6^A methylation changes in *fio1* loss of function mutants compared to wild type and to validate *FLC* methylation and stability in an unbiased fashion, we employed Nanopore direct RNA sequencing. This analysis showed that *fio1* mutants differ in the levels of transcriptome changes (Fig 7A). Furthermore, in Col-0 wild type plants, the majority (34.7%) of m^6^A methylations occurred in the GGACA element, followed by AGACT (27.2%), GGACT (22.9%) and GGACC (15.25) (Fig 7B). In summary, our work defined the *Arabidopsis* consensus m^6^A methylation site as RGACH, in which R represents A or G and H all nucleotides except G, which corresponds with the RRACH element that had previously been identified [2]. FIONA1 is a methyltransferase that adds methyl-groups to adenine bases of RNAs. Messenger-RNAs that are targets of FIO1 are therefore expected to be hypomethylated in a situation of lost or reduced FIO1 activity. Our direct RNA-sequencing approach yielded 74 genes that were hypomethylated in *fio1-1* mutants compared to wild type and 63 genes in *fio1-5* (S4 Table). Another recent direct RNA-sequencing study of the fio1-2 knock-down mutant revealed over 2000 hypomethylated transcripts in Arabidopsis [9]. The comparison with our datasets identified in total 28 hypomethylated transcripts that are detected in at least two mutants (Table 1). *FLC* expression was shown to be significantly reduced in both *fio1-1* and *fio1-5* mutants and meRIP-seq detected m^6^A methylation in the 3’UTR of *FLC* (Figs 4D, 5E and 6A–6C). In agreement with these latter results, direct RNA-sequencing confirmed that *FLC* mRNA is depleted in both *fio1-1* and *fio1-5* mutants (Fig 7D).

**Table 1:** Comparative analysis of hypomethylated transcripts in *fio1-1*, *fio1-2* and *fio1-5* relative to wild type Col-0.

Arabidopsis Gene Identifier (AGI)	Hypomethylated			Annotation
AT1G12840	*fio1-1*	*fio1-5*		DET3, ATVHA-C, ARABIDOPSIS THALIANA VACUOLAR ATP SYNTHASE SUBUNIT C, DE-ETIOLATED 3
AT1G19980	*fio1-1*	*fio1-5*		no symbol available
AT1G52040	*fio1-1*	*fio1-5*		MBP1, ATMBP, myrosinase-binding protein 1
AT1G52710	*fio1-1*	*fio1-5*		no symbol available
AT1G76730	*fio1-1*	*fio1-5*		COG0212, Clusters of Orthologous group 212
AT2G18050	*fio1-1*	*fio1-5*		HIS1-3, histone H1-3
AT2G40480	*fio1-1*	*fio1-5*		no symbol available
AT5G18790	*fio1-1*	*fio1-5*		no symbol available
AT5G56860	*fio1-1*	*fio1-5*		GNC, GATA21, GATA TRANSCRIPTION FACTOR 21
AT5G64860	*fio1-1*	*fio1-5*		AtDPE1, DPE1, disproportionating enzyme
AT1G50250	*fio1-1*	*fio1-2*		FTSH1, FTSH protease 1
AT1G52400	*fio1-1*	*fio1-2*		BGL1, ATBG1, BGLU18, A. THALIANA BETA-GLUCOSIDASE 1
AT1G63770	*fio1-1*	*fio1-2*		no symbol available
AT2G30520	*fio1-1*	*fio1-2*		RPT2, ROOT PHOTOTROPISM 2
AT2G47940	*fio1-1*	*fio1-2*		DEG2, DEGP2, EMB3117 | DEGP protease 2,
AT3G10060	*fio1-1*	*fio1-2*		no symbol available
AT3G51950	*fio1-1*	*fio1-2*		no symbol available
AT5G42650	*fio1-1*	*fio1-2*		CYP74A, AOS, DDE2, allene oxide synthase, DELAYED DEHISCENCE 2, CYTOCHROME P450 74A
AT5G66190	*fio1-1*	*fio1-2*		LFNR1, ATLFNR1, FNR1, leaf-type chloroplast-targeted FNR 1, LEAF FNR 1
AT1G67480	*fio1-2*	*fio1-5*		no symbol available
AT2G22990	*fio1-2*	*fio1-5*		SNG1, SCPL8, sinapoylglucose 1
AT4G19110	*fio1-2*	*fio1-5*		no symbol available
AT4G19160	*fio1-2*	*fio1-5*		no symbol available
AT5G25265	*fio1-2*	*fio1-5*		HPAT1, hydroxyproline O-arabinosylatransferase 1
AT5G57560	*fio1-2*	*fio1-5*		XTH22, TCH4, Touch 4, xyloglucan endotransglucosylase/hydrolase 22
AT2G01490	*fio1-1*	*fio1-2*	*fio1-5*	PAHX | phytanoyl-CoA 2-hydroxylase
AT2G28900	*fio1-1*	*fio1-2*	*fio1-5*	OEP16, OEP16-1, ATOEP16-L, ATOEP16-1, outer plastid envelope protein 16–1
AT4G08950	*fio1-1*	*fio1-2*	*fio1-5*	EXO, EXORDIUM

## Discussion

The precise timing of the floral transition is crucial for reproductive success. Premature as well as delayed flowering can result in seed dispersal at times where the offspring will be facing suboptimal conditions for survival and reproduction. This could either be due to the absence of pollinators or adverse environmental conditions. Therefore, a highly integrative network of transcription factors, but also epigenetic regulators, operate to ensure that flowering occurs in the most optimal conditions.

Methylation of mRNA is crucial for various functions within the cell. The m^6^A methylation of mRNA is an ancient molecular process and its disruption strongly compromises cellular functions. Strong reduction of the global m^6^A methylome early in plant development, as seen in mutants lacking the *METTL3*-homolog *MTA*, causes embryonic arrest [4]. Partial complementation of the *mta* mutant resulted in plants with compromised m^6^A levels that showed pleiotropic phenotypes such as reduced apical dominance and missing floral organs [24]. These latter results suggest that more subtle reductions of the global m^6^A levels are not detrimental to plant development. We provide further support of this by showing that the loss-of-function mutants of FIO1, a protein that is not essential for plant development, have only a subtle effect on the global m^6^A-methylome. However, CRISPR-induced mutants that caused larger genomic deletions in the *FIO1* gene (here *fio1-cr4* and *fio1-cr7*) showed a low-frequency seed abortion phenotype that resembles *mta* mutants (Fig 3G). Furthermore, in contrast to the effect that the loss of its homolog has on animal development, FIO1 is not essential and causes hypomethylation of specific transcripts. These hypomethylated mRNAs can then be stabilized, or destabilized, or mis-spliced. Affected transcripts that encode transcription factors or other regulators that are either mis-spliced of mis-methylated can subsequently induce alterations of circadian rhythms, cause changes in the production of hormones, or mis-regulation of other biological processes. It might be important to note that the pleiotropic phenotype of *fio1* mutant plants includes short stature, higher degree of shoot branching (Fig 3C) and a constitutive shade avoidance response (S5 Fig). A commonality of these phenotypes is that they relate to alterations in the levels of plant hormones, especially cytokinin and auxin. Wang et al. [21] found that *fio1* loss-of-function leads to an enrichment of hypomethylated genes associated with cytokinin signaling and ethylene response, potentially linking the phenotype we observe to the effect of FIO1 at the molecular level.

The precocious flowering phenotype is the most striking but *fio1* mutants additionally display a constitutive shade-avoidance phenotype, earlier senescence, and paler leaves [13]. In accordance with these phenotypes, our RNA-seq study revealed that several genes encoding circadian clock regulators and positive regulators of flowering time were upregulated in the *fio1* mutant background (e.g. *LHY*, *PIF4*). In contrast, several of the downregulated transcripts encoded transcription factors that repress flowering (Fig 4D and S1 Table).

Genetically, flowering is controlled by distinct pathways that interact at multiple levels to integrate inputs from all pathways. This integration ensures flowering occurs at the optimal time. The photoperiod pathway controls flowering in response to daylength and involves the B-Box zinc finger transcription factor CONSTANS (CO) which, in Arabidopsis, is stabilized at the end of long days [17]. CO positively regulates the expression of *FLOWERING LOCUS T* (*FT*) [25], encoding a mobile protein that travels to the shoot meristem to induce flowering [18]. *FIO1* acts partially through the photoperiod pathway and the early flowering phenotype of *fio1* mutants correlates with increased levels of both *CO* and *FT* mRNAs (S4 Fig) as well as increased levels of *SUPPRESSOR OF OVEREXPRESSION OF CONSTANS1* (*SOC1*) [9]. Our genetic interaction studies have shown that mutations in both *CO* and *FT* can partially suppress the early flowering effect of *fio1* mutants. Consistent with our findings, the *soc1* mutant has also been shown to partially suppress the early flowering phenotype of *fio1-2* mutant plants [9]. Taken together, these data support a model that assumes an indirect effect of the photoperiod pathway in the control of flowering by FIO1.

The spatial and temporal aspects of FIO1 function are currently unknown. It seems possible that depending on the developmental stage, the organ, or the age of the plant, that FIO1-executed m^6^A could differ and have different consequences downstream (directed mRNA modification versus changes in splicing patterns). Our analysis of the temporal changes in gene expression (Fig 4F) supports this notion. This could also explain contrasting results between papers, for example Xu et al. reported a consensus target sequence of YHm6AGA, which is significantly different from what has been reported here and by others, but their analysis was of 6-day old plants compared to 14- or 12-day old plants that were used here and in Wang et al., respectively.

Our RNA-sequencing data identified both up- and downregulated transcripts in *fio1* mutants compared to wild type. However, the overlap between the set of de-regulated transcripts identified in *fio1-2* mutants [9] is very limited. The latter fact can be attributed to the different types of mutations that were analyzed. While our study capitalized on mutant variants that are likely enzyme-dead and loss-of-function alleles, *fio1-2* is a T-DNA insertion line that still expresses *FIO1* mRNA, although at a lower level. Alternatively, the observed differences could be technical in nature, the result of either of the different sequencing approaches that were chosen or the growth conditions in which plants were cultivated. As described before, we have currently no knowledge on the spatial and temporal aspects of FIO1 function and differences in the circadian activity of FIO1 might also exist.

Our meRIP-sequencing approach further confirmed that FIO1 is likely not the main factor in the m^6^A modification of mRNAs but a more selective methyltransferase that modifies specific mRNAs. This assumption is supported by the finding that loss-of-function mutants are viable and able to produce mostly fertile offspring. Interestingly, despite the much higher number of differentially methylated transcripts in the *fio1-2* mutant [9], the comparison of the differentially hypomethylated transcripts compared to those in *fio1-1* and *fio1-5* (this study) produced only a very moderate overlap (Fig 7C). Again, this might be due to the application of different methods or an indication that the reduction of FIO1 activity affects the m^6^A methylome more strongly than does the complete loss. A recent paper characterized the effect of the loss of FIO1 function on global splicing patterns and profound changes were identified [16]. The authors relate these changes to defects in U6 snRNA m^6^A modification, rather than being a direct consequence of loss of FIO1 activity.

The analysis of the m^6^A consensus in *fio1-2* identified the YHAGA motif, which is significantly different to the RRACH motif that has been described in both plants and animals [2, 26], and to the RGACH consensus sequence that we identify in this work (Fig 7B) and the motifs identified by Parker et al [16].

Detailed analysis of specific transcripts that are differentially methylated and differentially expressed led us to the flowering regulator *FLC*. Regardless of whether the contribution of *FLC* methylation contributes only marginally to the early flowering response of *fio1* mutants, our work unequivocally demonstrates that FIO1 is the m^6^A-methyltransferase that methylates the 3’UTR of *FLC* mRNA. We show that the failure to methylate *FLC* mRNA targets it for rapid degradation, hence the absence of *FLC* mRNA in *fio1* mutants (Fig 6). Further characterization of the relationship between FIO1 and the biology of *FLC* will lead to insights into the function of its 3’-end methylation.

Our analyses focused on the role of methylation of mRNAs and the impact on the regulation of flowering. We cannot rule out confounding effects that the loss of *FIO1* may have on the methylation and regulation of the non-coding transcriptome. Such effects and changes in splicing patterns might also greatly contribute to the pleiotropic phenotype of *fio1* mutant plants and further characterization is needed to shed light on these processes.

## Methods

### Plant materials and growth conditions

*Arabidopsis thaliana* genotypes used in the study were, if not otherwise stated, in the Columbia Col-0 background. Double and triple mutant plants, such as *fio1 co-sail*, *fio1 ft10* and *fio1 miP1a miP1b* were generated by genetic crossing. For flowering experiments, seeds were stratified 48 h at 4°C, and grown on soil in a plant growth chamber under long daylight conditions (16 h light / 8 h dark), or short daylight conditions (8 h light / 16 h dark) at 22°C day / 20°C night. Flowering time was measured by counting the number of rosette leaves at the bolting stage.

For RNA-seq, MeRIP-seq, Nanopore direct sequencing, and qPCR, 14-day old seedlings were collected. Seeds were sterilized in 70% ethanol and sown on 1/2 Murashige and Skoog (MS) medium plates with 0.8% agar and kept at 4°C for 48 hours in darkness for stratification and then grown at (22°C day / 20°C night) and 70% humidity under long daylight conditions (16 h light / 8 h dark).

Loss-of-function mutants of *fio1* were generated using the CRISPR/Cas9 vector pKI1.1R, containing the Cas9 expression cassette (RPS5Ap::Cas9:HspT), a sgRNA expression cassette (U6.26p::AarI_site:sgRNA) and, for selection the RFP expression cassette (OLE1p::OLE1:TagRFP). Single-guide RNAs (sgRNAs) were designed using the web tool CRISPR-P v 2.0 [27]. Vectors with sgRNAs were generated according to the published description [28]. To create mutants with deletions, two to three Agrobacterium strains GV3101 pMD90 with different sgRNAs (S3 Table) were pooled and transformed into wild type plants via floral dip. RFP-positive seeds were selected using a Leica MZFLIII stereomicroscope equipped with RFP filters. Deletions were detected by PCR based sequencing.

### Mapping-by-sequencing

91.99% sequenced reads were mapped by Bowtie2 (v2.1.0)[29] using the TAIR9 genome assembly and TAIR10 annotation from Phytozome v10.3 (phytozome.org). SNP calling was performed using samtools and BCFtools (v0.1.19)[30, 31]. 1118 (Chr1: 203, Chr2: 194, Chr3: 247, Chr4: 189, Chr5: 285) background corrected EMS-induced SNP markers were identified by SHOREmap[32] (v3.2) using standard settings. Finally, the mutations indicated a mapping interval of 7 Mb Kb on chromosome 2, containing 84 mutations. The trend line is the average of all SNP allele frequencies in a sliding window (size: 2,500 Kb; step: 100 Kb). Mapping-by-sequencing data has been deposited in NCBI’s Gene Expression Omnibus under GEO Series accession no. GSE171924.

### FIO1 homology modeling

The methyltransferase domain of FIONA1 (UniProt accession code F4IGH3, residues 1–333) was modelled with Phyre2 (http://www.sbg.bio.ic.ac.uk/phyre2) using the Intensive modelling mode. The resulting homology model was aligned against the human crystal structure of the human FIONA1 homologue, METTL16 (PDB ID: 6DU4) for structural analysis.”

### mRNA sequencing analysis

For RNAseq analysis, we collected two biological replicates of 14 day-old wild type (Col-0), *fio1-1*, *fio1-5* seedlings. Total RNA was extracted from 100 *A*. *thaliana* seedlings for each line grown on a ½ MS agar plate using the Spectrum Plant Total RNA Kit (Sigma-Aldrich) following the manufacturer’s instructions. Total RNA was treated with DNAase I (RapidOut DNA Removal Kit, Thermo Scientific) according to the manufacturer’s instructions. Sequencing library preparation and sequencing on an Illumina HiSeq4000 instrument was performed by Novogene (Hongkong). About 3.7 Gb high-quality 150-bp paired-end reads were generated from each library. FastQC (Galaxy Version 0.72 + galaxy1) was initially run to assess the overall quality of all sample reads. Poor quality bases and adapters were filtered out using Trim Galore (Galaxy Version 0.6.3). The quality-filtered reads were aligned to the *Arabidopsis thaliana* reference genome (TAIR10) using HISAT282 (Version 2.1.0 + Galaxy4) with default parameters. HTseq (Galaxy Version 0.9.1) software was used to count the number of raw reads mapped to each of the genes. Differential expression analysis was performed with four analytical methods, DEseq 2 (Galaxy Version 2.11.40.6+galaxy1), edgeR (Galaxy Version 3.24.1+galaxy1), Limma-voom (Galaxy Version 3.38.3+galaxy3) and Limma-trend (Galaxy Version 3.38.3+galaxy3). All four statistical methods gave similar overall conclusions. We selected the most conservative results (Limma-voom; false discovery rate (FDR) = 0.05) for further investigation. Significance testing was performed using the Benjamini-Hochberg method[33]. Genes showing an absolute value of log2 FC (fold change; *fio1* mutant / WT) ≥ 1.0 and adjusted P-value (false discovery rate; FDR) < 0.05 were considered as differentially expressed genes. RNAseq data generated in this study has been deposited in NCBI’s Gene Expression Omnibus under GEO Series accession no. GSE171926.

### m6A RNA Immunoprecipitation sequencing (MeRIP-seq) and data analysis

MeRIP-seq was performed as described before[22] with modifications. Briefly, total RNA was extracted from 14 day-old Arabidopsis thaliana seedlings using the Spectrum Plant Total RNA Kit (Sigma-Aldrich) and treated with DNAase I (RapidOut DNA Removal Kit, Thermo Scientific). 300 μg of total RNA was mixed with 10×Fragmentation buffer (1 M Tris-HCl pH = 7.0, 1 M ZnCl2) and placed at 94°C for 5 min then snap cooled on ice for 5 minutes. The volume of fragmented RNA was then adjusted to 755 μl with RNase-free water. Next, 10 μL RNasin Plus RNase inhibitor (Promega, cat. no. N2611), 10 μL Ribonucleoside vanadyl complexes (RVC; 200 mM; Sigma-Aldrich, cat. no. R3380), 200 μL 5×IP buffer (50 mM Tris-HCl, 750 mM NaCl and 0.5% (vol/vol) Igepal CA-630), and 25 μL of m6A antibody (Synaptic Systems, cat. no. 202 003) were added to samples and samples were rotated at 4°C for 2 hours. After 2 hours, pre-blocked Protein A Dynabeads (Thermo Fisher, 1001D) was added to the RNA samples and rotated for an additional 2 hours at 4°C. After 2 hours, Dynabeads were pelleted using a magnetic stand and washed three times with 1 mL 1×IP buffer. RNA was eluted from Dynabeads by adding 98 μL elution buffer (20 mM Tris-HCl pH 7.5, 300 mM sodium acetate, 2 mM EDTA, 0.25% SDS), 2 μL of proteinase K (Thermo Fisher, AM2546) and then shaking for 1 hour at 37°C. All samples were precipitated using 3 M sodium acetate (pH 5.2) and 2.5 volumes of 100% ethanol and kept at -80°C overnight. Libraries were prepared using NEBNext Multiplex Small RNA Library Prep Set for Illumina (New England BioLabs, E7300S) according to the manufacturer’s instructions. Novogene (Beijing) performed sequencing on an Illumina HiSeq4000 instrument. About 3.0 Gb high-quality 150-bp paired-end reads were generated from each library. FastQC (Galaxy Version 0.72 + galaxy1) was initially run to assess the overall quality of all sample reads. Poor quality bases and adapters were filtered out using Trim Galore (Galaxy Version 0.6.3). The quality-filtered reads were aligned to the *A*. *thaliana* reference genome using HISAT2 (Version 2.1.0 + Galaxy4) with default parameters. To identify regions in which m6A modifications occurred, MACS [23] was used to call peaks on aligned files. The peaks showing an absolute value of log2 FC (fold change; *fio1* mutant / WT) ≥ 0.5 and RPM ≥ 5 were considered as differentially modified peaks. MeRIPseq data generated in this study has been deposited in NCBI’s Gene Expression Omnibus under GEO Series accession no. GSE171928.

### mRNA stability measurements

mRNA stability measurement assay was performed as previously described [34] with modification. Briefly, 12-day-old *Arabidopsis* seedlings grown on 1/2 MS medium were transferred to 10-cm Petri dishes containing 1/2 MS liquid medium at ZT13. After 30 min incubation, 0.2 mM actinomycin D was added to the buffer. The tissues were collected at 1 h after the transcription inhibitor was added; these samples are referred to as 0 h samples. The 2 h and 4 h samples were collected and immediately frozen in liquid nitrogen. The total RNA was isolated from these tissues, and the remaining mRNA levels were quantified by RT-qPCR with gene-specific qPCR primers. 18S RNA was used as the internal control, and *AT2G07689* was used as a negative control [35]. Primers used for qPCR: AT2G07689-qF;CATTACGGCAAACCCGTGTC | AT2G07689-qR;GGCTAACGGGGGTATTCCTG | FLC-qF;GAGAACAAAAGTAGCCGACAAGTC | FLC-qR;GGATGCGTCACAGAGAACAGA | 18s-qF;GCGGCTTAATTTGACTCAACACG | 18s-qR;CCTGTTATTGCCTCAAACTTCC

Primers for FA-RIP-qPCR, m6A-IP-qPCR and mRNA stability assay: FLC-IP-qPCR-F;CTCCCACTACTTAATTAGCCACCTTA | FLC-IP-qPCR-R;CCCTTATCAGCGGAATAATTACATATC

### Nanopore direct RNA sequencing

Total RNA was isolated as described above for mRNA-seq and direct RNA sequencing libraries were prepared by CD genomics using the Oxford Nanopore DRS protocol (SQK-RNA002, Oxford Nanopore Technologies). Samples were loaded into the Nanopore R9.4 sequencing micro-array and sequenced for 48–72 hrs using the PromethION sequencer (Oxford Nanopore Technologies). Read quality assessment, base calling and adapter trimming was carried out with the Guppy software (version 3.2.6). Nanofilt (version 2.7.1) was then used to remove low quality reads (Q-value < 7) and short-length reads (<50 bp). The clean reads were subsequently corrected using Fclmr2 (version 0.1.2). Minimap2 (version 2.17-r941) was used to map the clean reads to the *A*. *thaliana* genome and the alignment ratio of clean reads to the reference genes was calculated using Samtools (version 1.10). To identify m6A sites, the Tombo software de novo model together with MINES was used for calculation. Methylkit software was then used to analyze differential methylation sites (DML). Logistic regression test was used to detect differential methylation sites. Nanopore direct RNA-sequencing data has been deposited in NCBI’s Gene Expression Omnibus under GEO Series accession no. GSE212766.

### RNA m^6^A immunoprecipitation RT-qPCR

Quantitative real-time PCR was performed to assess relative abundance of m6A RNA in the RIP samples. 300 μg total RNA was adjusted the volume to 1000 μl with 5×IP buffer (50 mM Tris-HCl, 750 mM NaCl and 0.5% (vol/vol) Igepal CA-630) and RNase-free water and incubated with 10 μg m6A antibody (Synaptic Systems, cat. no. 202 003, Goettingen, Germany). The mixture was rotated at 4°C for 2 h, then pre-blocked and washed Dynabead Protein A (Thermo Fisher, 1001D) were added and the mixture rotated for an additional 2 h at 4°C. After washing with IP buffer containing Ribonucleoside vanadyl complexes (RVC, Sigma, R3380-5ML) three times, the m6A IP RNA was eluted with 98 μL elution buffer (20 mM Tris-HCl pH 7.5, 300 mM sodium acetate, 2 mM EDTA, 0.25% SDS). 2 μL of proteinase K (Thermo Fisher, AM2546) was added and the RNA incubated for 1 hour at 37°C with gentle shaking. All samples were precipitated using 3 M sodium acetate (pH 5.2) and 2.5 volumes of 100% ethanol and kept at -80°C overnight. cDNA was synthesized by iScrip cDNA Synthesis Kit (Bio-Rad). qPCR analyses was done with Ultra SYBR Mixture with ROX (CWBIO) on a CFX384 Touch Real-Time PCR Detection System (Bio-Rad). qRT- PCR primers that were used to amplify *FLC* were: flc_qF: AGCCAAGAAGACCGAACTCA and flc_qR: TTTGTCCAGCAGGTGACATC.

## Supporting information

S1 Fig Analysis of *fio1-5*, a CRISPR-induced mutation in *FIO1*. **(A)** Phenotype of *fio1-5* compared to the Col-0 wildtype when grown in LD conditions. **(B)** Determination of flowering by counting the number of rosette leaves (RLN = rosette leaf number) at the bolting stage in LD. Plotted are average leaf number +/- SD, ***p = <0.001, N = 10–14. **(C)** Nucleotide alignment showing the CRISPR-induced genomic deletion found in *fio1-5*. Gene model on top shows the relative positions of all three *fio1* mutations.
(PDF)

S2 Fig Analysis of FIO1 methyltransferase domain mutants based on homology model. The three mutants were mapped to the homology model of FIO1 (see Materials and Methods). The *fio1-1* mutation involved the loss of five amino acids highlighted in pink, including the loss of a potential hydrogen bond between the threonine and asparagine. The *sum8* mutation changes the serine (orange), which normally hydrogen bonds to a tryptophan, into an asparagine. The resulting larger side-chain of asparagine is unlikely to be accommodated in the constrained protein interior, leading to changes in the protein structure and loss of function. The *fio1-5* mutation involves a large deletion (orange) and missense mutations (light cyan) in a partially buried alpha helix, which are very likely to disrupt protein folding and function.
(PDF)

S3 Fig Flowering time analysis CRISPR-induced mutations in *FIO1*. Plants were grown in long day conditions (16h light, 8 hour dark) and the number of leaves were counted at the bolting stage. Depicted is the average +/- standard deviation. N = 10.
(PDF)

S4 Fig FIONA1 acts partially independent of the photoperiod pathway to repress flowering. **(A)** and **(B)** Quantification of *CO* and *FT* in Col-0, *fio1-1* and *fio1-5* by qRT-PCR. Values are the means ±SD. N = 4. * P ≤ 0.01. **(C)** Phenotypes of *miP1a miP1b*, *fio1-1*, *fio1-5*, *miP1a miP1b fio1-5*, *co-sail*, *co-sail fio1-1*, *co-sail fio1-5*, *ft10*, *ft10 fio1-1*, *ft10 fio1-5* and determination of flowering time by counting the number of rosette leaves at bolting compare to wild type, under long day conditions. RLN = number of rosette leaves at the bolting stage. Values are the means ±SD. N = 10 to 20. One-way ANOVA was carried out to test significance, **P ≤ 0.005, ***P≤ 0.001. **(D)** Phenotypes of *miP1a miP1b*, *fio1-1*, *fio1-5*, *miP1a miP1b fio1-5*, *co-sail*, *co-sail fio1-1*, *co-sail fio1-5*, *ft10*, *ft10 fio1-1*, *ft10 fio1-5* and determination of flowering time by counting the number of rosette leaves at bolting compare to wild type, under short day conditions. RLN = number of rosette leaves at the bolting stage. Values are the means ±SD. N = 10 to 12. One-way ANOVA was carried out to test significance, ***P≤ 0.001.
(PDF)

S5 Fig Analysis of hypocotyl elongation in response to elevated far-red levels (shade avoidance response). Hypocotyl length of Col-0 wildtype and *fio1-1* and *fio1-5* mutants grown in either white light conditions or in far-red light enriched white light conditions (+FR). The *fio1* mutants show a hypersensitivity response with increase hypocotyls in white light and even longer hypocotyls in shade conditions.
(PDF)

S1 Table DEGs identified in *fio1-1* and *fio1-5* by RNAseq. (XLSX)

S2 Table Methylation peakes identified by MeRIP-seq. (XLSX)

S3 Table Oligonucleotide sequences. (XLSX)

S4 Table Hypomethylated transcripts identified in *fio1-1* and *fio1-5*. (XLSX)
