# Supplementary material for: FIONA1-mediated methylation of the 3’UTR of FLC affects FLC transcript levels and flowering in Arabidopsis
Source: PLoS Genet. 2022 Sep 27;18(9):e1010386. doi: 10.1371/journal.pgen.1010386 (PMC9543952; doi:10.1371/journal.pgen.1010386)
Supplement: S1 Fig — (A) Phenotype of fio1-5 compared to the Col-0 wildtype when grown in LD conditions. (B) Determination of flowering by counting the number of rosette leaves (RLN = rosette leaf number) at the bolting stage in LD. Plotted are average leaf number +/- SD, ***p = <0.001, N = 10–14. (C) Nucleotide alignment showing the CRISPR-induced genomic deletion found in fio1-5. Gene model on top shows the relative positions of all three fio1 mutations. (PDF) [file pgen.1010386.s001.pdf]

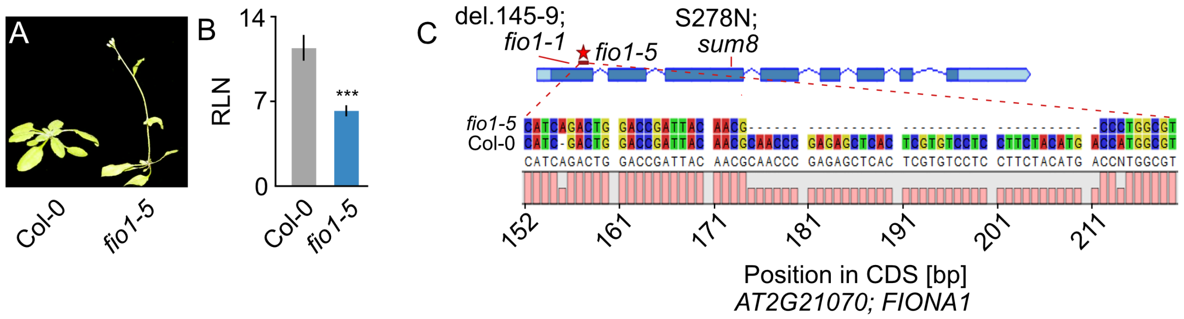

Figure S1 – CRISPR-induced mutation in *FIO1*.
(A) Phenotype of *fio1-3* compared to the Col-0 wildtype when grown in LD conditions.
(B) Determination of flowering by counting the number of rosette leaves (RLN = rosette leaf number) at the bolting stage in LD. Plotted are average leaf number +/- SD, ***p=<0.001, N=10-14.
(C) Nucleotide alignment showing the CRISPR-induced genomic deletion found in *fio1-5*. Gene model on top shows the relative positions of all three *fio1* mutations.
