# Supplementary material for: FIONA1-mediated methylation of the 3’UTR of FLC affects FLC transcript levels and flowering in Arabidopsis
Source: PLoS Genet. 2022 Sep 27;18(9):e1010386. doi: 10.1371/journal.pgen.1010386 (PMC9543952; doi:10.1371/journal.pgen.1010386)
Supplement: S2 Fig — The three mutants were mapped to the homology model of FIO1 (see Materials and Methods). The fio1-1 mutation involved the loss of five amino acids highlighted in pink, including the loss of a potential hydrogen bond between the threonine and asparagine. The sum8 mutation changes the serine (orange), which normally hydrogen bonds to a tryptophan, into an asparagine. The resulting larger side-chain of asparagine is unlikely to be accommodated in the constrained protein interior, leading to changes in the protein structure and loss of function. The fio1-5 mutation involves a large deletion (orange) and missense mutations (light cyan) in a partially buried alpha helix, which are very likely to disrupt protein folding and function. (PDF) [file pgen.1010386.s002.pdf]

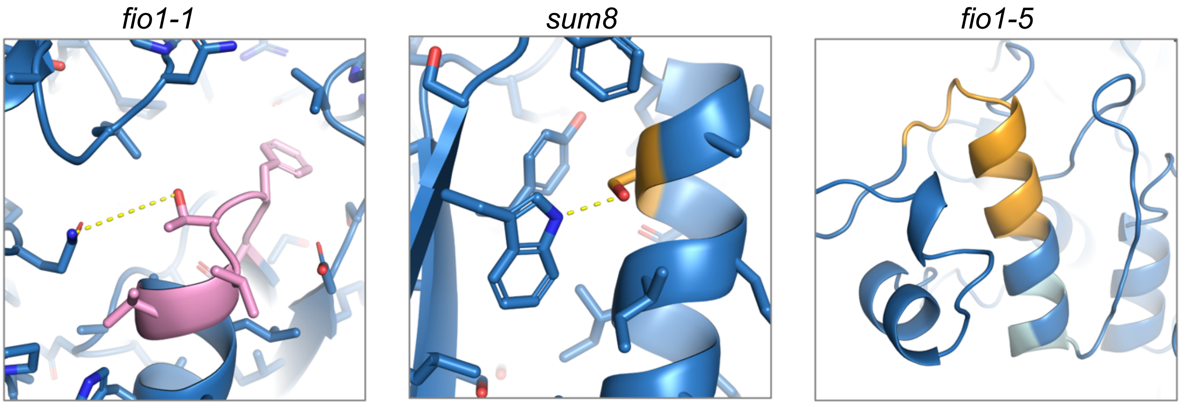

Supplementary Figure S2 – Analysis of FIO1 methyltransferase domain mutants based on homology model.

The three mutants were mapped to the homology model of FIO1 (see Materials and Methods). The *fio1-1* mutation involved the loss of five amino acids highlighted in pink, including the loss of a potential hydrogen bond between the threonine and asparagine. The *sum8* mutation changes the serine (orange), which normally hydrogen bonds to a tryptophan, into an asparagine. The resulting larger side-chain of asparagine is unlikely to be accommodated in the constrained protein interior, leading to changes in the protein structure and loss of function. The *fio1-5* mutation involves a large deletion (orange) and missense mutations (light cyan) in a partially buried alpha helix, which are very likely to disrupt protein folding and function.
