# Supplementary material for: FIONA1-mediated methylation of the 3’UTR of FLC affects FLC transcript levels and flowering in Arabidopsis
Source: PLoS Genet. 2022 Sep 27;18(9):e1010386. doi: 10.1371/journal.pgen.1010386 (PMC9543952; doi:10.1371/journal.pgen.1010386)
Supplement: S3 Fig — Plants were grown in long day conditions (16h light, 8 hour dark) and the number of leaves were counted at the bolting stage. Depicted is the average +/- standard deviation. N = 10. (PDF) [file pgen.1010386.s003.pdf]

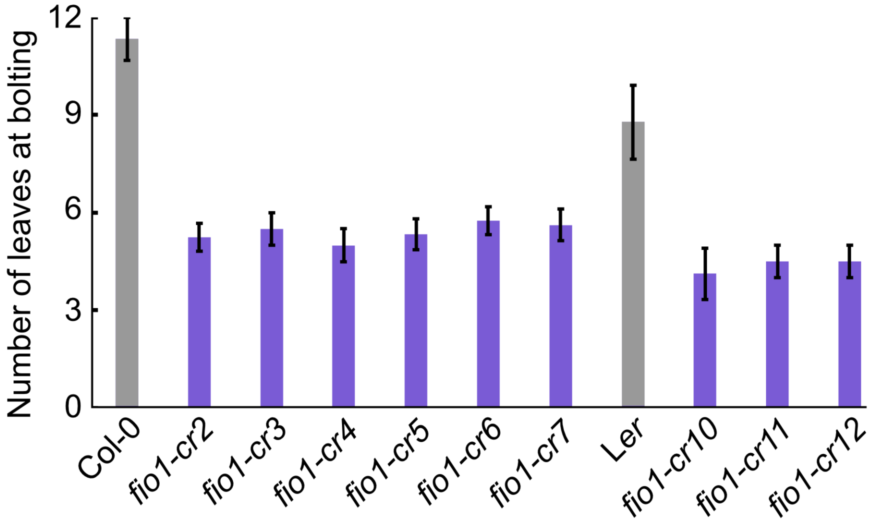

Supplementary Figure S3 – Flowering time analysis CRISPR-induced mutations in *FIO1*. Plants were grown in long day conditions (16h light, 8 hour dark) and the number of leaves were counted at the bolting stage. Depicted is the average +/- standard deviation. N=10
