# Supplementary material for: FIONA1-mediated methylation of the 3’UTR of FLC affects FLC transcript levels and flowering in Arabidopsis
Source: PLoS Genet. 2022 Sep 27;18(9):e1010386. doi: 10.1371/journal.pgen.1010386 (PMC9543952; doi:10.1371/journal.pgen.1010386)
Supplement: S4 Fig — (A) and (B) Quantification of CO and FT in Col-0, fio1-1 and fio1-5 by qRT-PCR. Values are the means ±SD. N = 4. * P ≤ 0.01. (C) Phenotypes of miP1a miP1b, fio1-1, fio1-5, miP1a miP1b fio1-5, co-sail, co-sail fio1-1, co-sail fio1-5, ft10, ft10 fio1-1, ft10 fio1-5 and determination of flowering time by counting the number of rosette leaves at bolting compare to wild type, under long day conditions. RLN = number of rosette leaves at the bolting stage. Values are the means ±SD. N = 10 to 20. One-way ANOVA was carried out to test significance, **P ≤ 0.005, ***P≤ 0.001. (D) Phenotypes of miP1a miP1b, fio1-1, fio1-5, miP1a miP1b fio1-5, co-sail, co-sail fio1-1, co-sail fio1-5, ft10, ft10 fio1-1, ft10 fio1-5 and determination of flowering time by counting the number of rosette leaves at bolting compare to wild type, under short day conditions. RLN = number of rosette leaves at the bolting stage. Values are the means ±SD. N = 10 to 12. One-way ANOVA was carried out to test significance, ***P≤ 0.001. (PDF) [file pgen.1010386.s004.pdf]

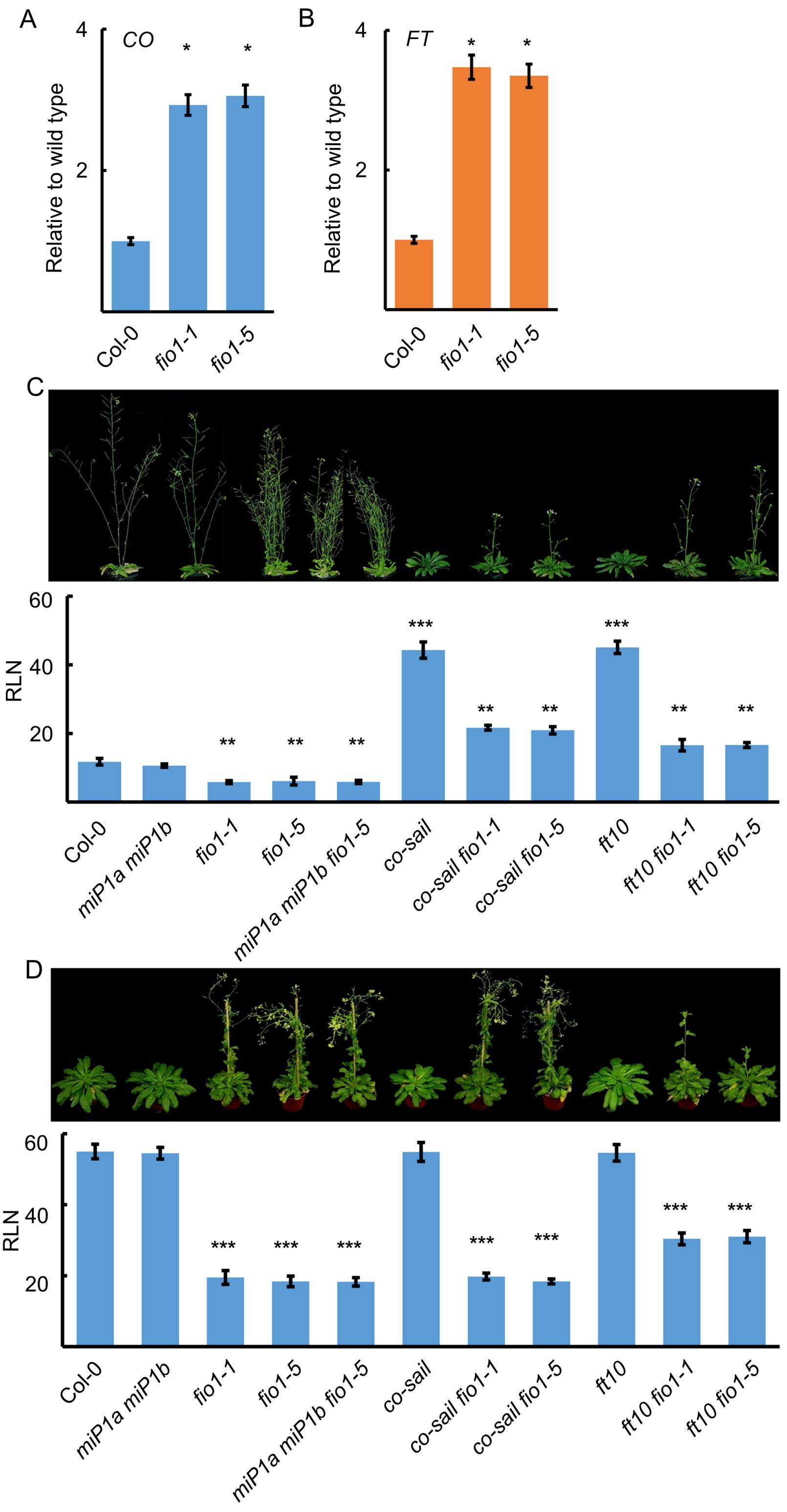

Figure S4 – FIONA1 acts partially independent of the photoperiod pathway to repress flowering.
(A) and **(B)** Quantification of *CO* and *FT* in Col-0, *fio1-1* and *fio1-5* by qRT-PCR. Values are the means ±SD. N = 4. * P ≤ 0.01.
(C) Phenotypes of *miP1a miP1b*, *fio1-1*, *fio1-5*, *miP1a miP1b fio1-5*, *co-sail*, *co-sail fio1-1*, *co-sail fio1-5*, *ft10*, *ft10 fio1-1*, *ft10 fio1-5* and determination of flowering time by counting the number of rosette leaves at bolting compare to wild type, under long day conditions. RLN = number of rosette leaves at the bolting stage. Values are the means ±SD. N = 10 to 20. One-way ANOVA was carried out to test significance, **P ≤ 0.005, ***P≤ 0.001.
(D) Phenotypes of *miP1a miP1b*, *fio1-1*, *fio1-5*, *miP1a miP1b fio1-5*, *co-sail*, *co-sail fio1-1*, *co-sail fio1-5*, *ft10*, *ft10 fio1-1*, *ft10 fio1-5* and determination of flowering time by counting the number of rosette leaves at bolting compare to wild type, under short day conditions. RLN = number of rosette leaves at the bolting stage. Values are the means ±SD. N = 10 to 12. One-way ANOVA was carried out to test significance, ***P≤ 0.001.
