# Supplementary material for: FIONA1-mediated methylation of the 3’UTR of FLC affects FLC transcript levels and flowering in Arabidopsis
Source: PLoS Genet. 2022 Sep 27;18(9):e1010386. doi: 10.1371/journal.pgen.1010386 (PMC9543952; doi:10.1371/journal.pgen.1010386)
Supplement: S5 Fig — Hypocotyl length of Col-0 wildtype and fio1-1 and fio1-5 mutants grown in either white light conditions or in far-red light enriched white light conditions (+FR). The fio1 mutants show a hypersensitivity response with increase hypocotyls in white light and even longer hypocotyls in shade conditions. (PDF) [file pgen.1010386.s005.pdf]

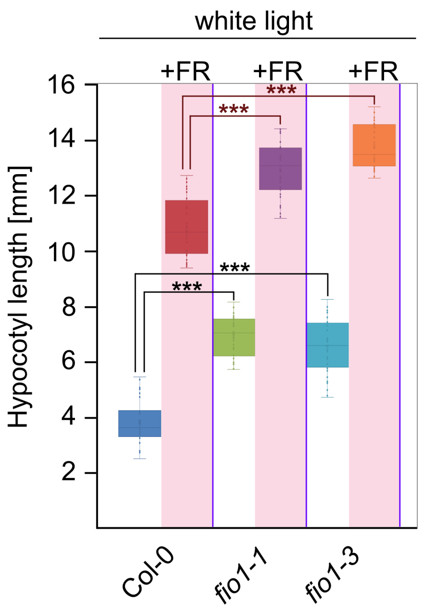

Supplementary Figure S5 – Analysis of hypocotyl elongation in response to elevated far-red levels (shade avoidance response). Hypocotyl length of Col-0 wildtype and *fio1-1* and *fio1-5* mutants grown in either white light conditions or in far-red light enriched white light conditions (+FR). The *fio1* mutants show a hypersensitivity response with increase hypocotyls in white light and even longer hypocotyls in shade conditions.
